# Pemetrexed Maintenance Therapy Following Bevacizumab-Containing First-Line Chemotherapy in Advanced Malignant Pleural Mesothelioma: A Case Report and Literatures Review

**DOI:** 10.1097/MD.0000000000003351

**Published:** 2016-04-08

**Authors:** Xu-Quan Jing, Lei Zhou, Xin-Dong Sun, Jin-Ming Yu, Xue Meng

**Affiliations:** From the Departments of Radiation Oncology (X-QJ, X-DS, J-MY, XM) and Orthopedics (LZ), Shandong Cancer Hospital and Institute, Jinan, Shandong, China.

## Abstract

Malignant pleural mesothelioma (MPM) is a lethal disease with poor prognosis. The combination of cisplatin and pemetrexed has been confirmed as the standard of care for nonoperable MPM. Data have shown that the adoption of pemetrexed maintenance therapy (PMT) following first-line treatment appears extremely promising.

We describe a 57-year-old man diagnosed as advanced MPM. We treated this patient with PMT after first-line cisplatin-based bevacizumab-containing chemotherapy and residual tumor disappeared after 6 course of PMT. A perfect response and a long progression-free survival (PFS) were reached with tumor mass disappearing and 14 months duration of PFS.

This case suggests that adding bevacizumab to standard first-line chemotherapy is feasible and that PMT could be promising and useful for treating advanced MPM. We further entail a review of the literature on the first-line treatment, continuation maintenance therapy, switch maintenance therapy, and second-line treatment of patients with advanced MPM.

## INTRODUCTION

Malignant pleural mesothelioma (MPM) is a locally aggressive, usually fatal malignancy stemming from the pleural mesothelial surfaces, with a median survival of 4 to18 months after diagnosis.^1^ MPM incidence has continued to increase worldwide,^2^ and it is not expected to drop until sometime between 2015 and 2030.^3^ Following the results of a large phase III trial, the combination of cisplatin and pemetrexed has been established as the standard of care (SOC) for advanced MPM.^4^ However, nearly all MPM patients progress during or after first-line treatment. So, progression-free survival (PFS) was not satisfied for patients with MPM.

The efficacy of treatment of cisplatin-containing chemotherapy as SOC reaches a platform. It is difficult to further improve the prognosis of advanced MPM with mere standard combination schedule. Whether it can promote PFS or survival to add additional drug such as target therapy to first-line SOC? Besides, the role of pemetrexed maintenance therapy (PMT) in responding or stable patients with MPM after receiving first-line treatment has not been confirmed and the question of the benefits of PMT for MPM remains open. Is it reasonable and flexible to incorporate this new strategy before disease progression?

We therefore report a case of advanced MPM treated with a combinational revenue of cisplatin, pemetrexed, and bevacizumab as first-line care and subsequent strategy of PMT with breaking though the SOC for advanced MPM, expecting to further improve PFS. The patient presented good response after 6 courses PMT and achieved a strikingly long duration of PFS.

## CASE PRESENTATION

**FIGURE 1:**
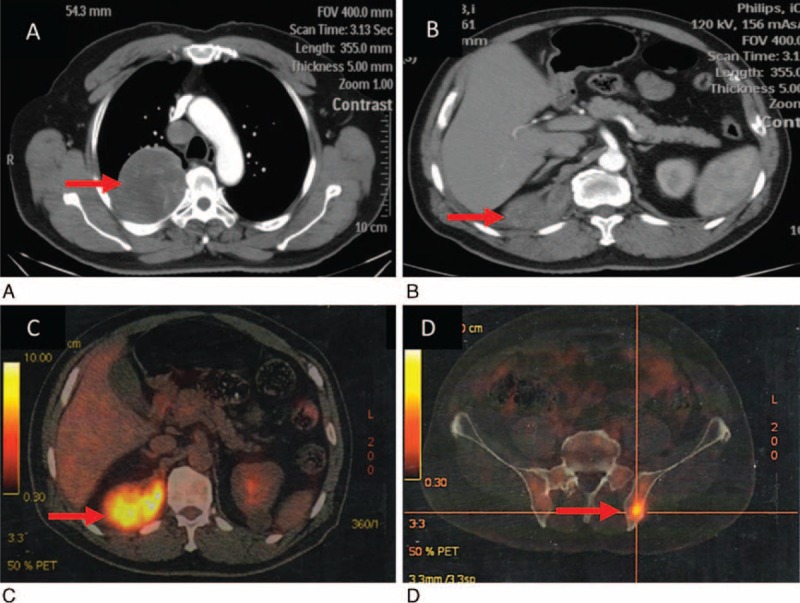
(A) Computed tomography (CT) demonstrated a large right-sided pleural mass with invasion to superior lobe of right lung. (B) CT showed nodule lying in the costophrenic angle. (C) Fluorodeoxyglucose (^18^F-FDG) position-emission tomography (PET)-CT after surgery revealed the residual disease and metastasis of bone (D).

A 57-year-old man with a 5-year history of smoking from 30 years of his age was referred to community hospital and complained of right chest pain for about 1 month. The patient classified his pain as 2/10, which usually was worse with activity. Physical examination suggested no significant abnormalities. Laboratory findings were within normal range, except for the neuron-specific enolase (NSE) level of 48.04 ng/mL (normal range, 0–24 ng/mL) in the serum. His computed tomography (CT) scan of the chest with contrast revealed a large right-sided pleural mass and other nodules lying in the costophrenic angle, suggestive of pleural malignancy (Figure 1A and B). Besides, the enlarged right lower paratracheal lymph nodes (4R) was seen in CT images. Bone scintigraphy showed no positive signs. The International Mesothelioma Interest Group clinical stage was III (T_3_N_2_M_0_).

**FIGURE 2:**
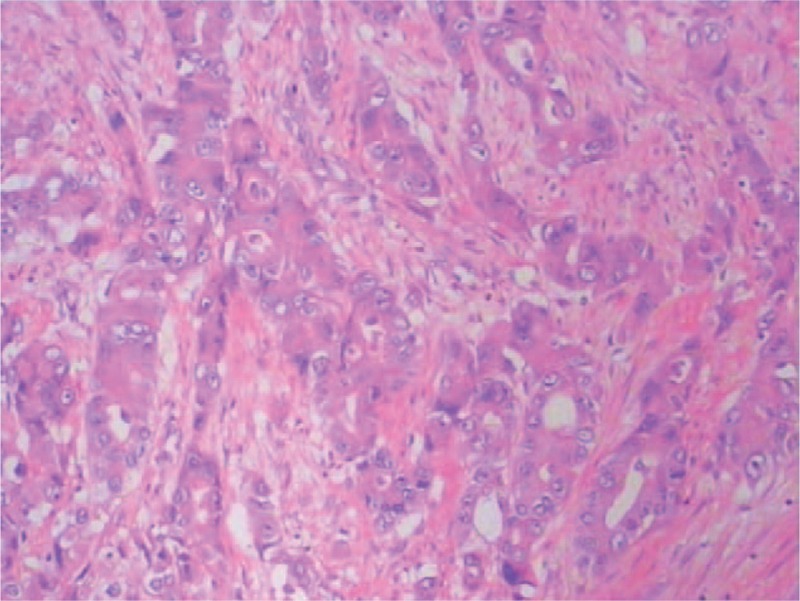
Pathology of tissues confirmed malignant pleural mesothelioma (MPM). H–E staining, ×400. H–E = hematoxylin and eosin.

Subsequently, a pleurectomy/decortication was performed. However, a mass could not be resected because of invasion to inferior vena cava incidentally found in surgery. After surgery, the resected specimens were sent for pathological and immunohistochemistry (IHC) analysis. The pathological diagnosis of the specimens was MPM (Figure 2) and confirmed the metastasis of 4R lymph node. Results of IHC staining were that WT-1 was positive and that Calretinin, CK7, EMA, TTF-1, Vimentin, CK, CK5, and OCT-4 were negative, respectively. And the IHC also suggested that Ki-67 ranged from 50% to 75%. There was no additional therapy after surgery. Two weeks after surgery, fluorodeoxyglucose (^18^F-FDG) position-emission tomography (PET)-CT was performed and revealed the residual disease and metastasis of bone (Figure 1C and D).

**FIGURE 3:**
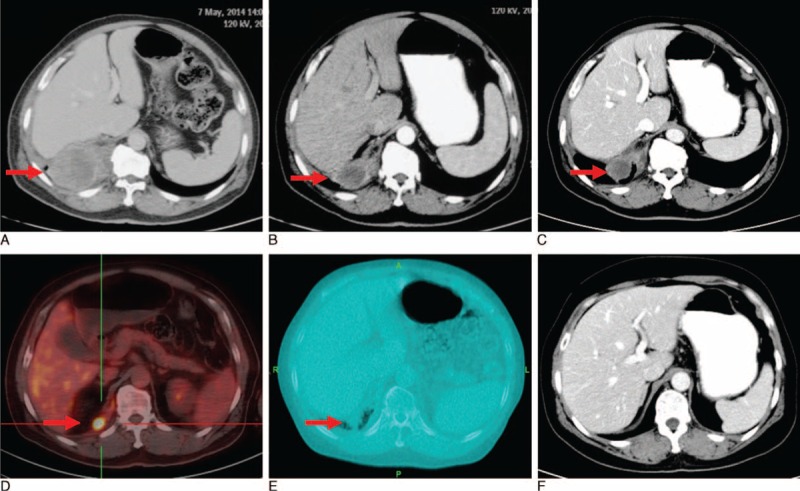
(A) Residual disease was also demonstrated by CT before the initial chemotherapy. (B) After 2 cycles of fist-line chemotherapy, the disease was partial response. (C) After 4 cycles of fist-line chemotherapy, the disease was partial response. (D) After 6 cycles of fist-line chemotherapy, the disease was stable. (E) The disease remained stable after radiotherapy. (Note: this picture is captured by megavoltage CT (MVCT) in tomotherapy system at the time of the last irradiation.) (F) After 6 course of pemetrexed maintenance therapy (PMT), the residual mass disappeared. CT = computed tomography.

The patient was transferred to our hospital (a tertiary care hospital) for further treatment. His CT scan with contrast showed tumor residue (Figure 3A). Bone metastasis was confirmed by magnetic response image. So, the clinical stage updated to stage IV. After multidisciplinary discussion, the combination of cisplatin (75 mg/m^2^), pemetrexed (500 mg/m^2^), and bevacizumab (7.5 mg/kg) as first-line chemotherapy was administered every 21 days for 6 cycles. Due to no pain of bone metastasis areas, only Ibandronate sodium in absence of radiotherapy was given monthly for skeletal-related events. Response evaluation was performed after every 2 cycles of chemotherapy according to the modified Response Evaluation Criteria in Solid Tumors criteria.^5^ The response after 2 courses, 4 courses, 6 courses was partial response (PR), PR, PR, respectively (Figure 3B–D). Taking tumor residue into consideration, the adjuvant tomotherapy was given to the residual mass with total dose 55 Gy in 31 fractions. The disease response stayed PR after radiotherapy (Figure 3E).

**FIGURE 4:**
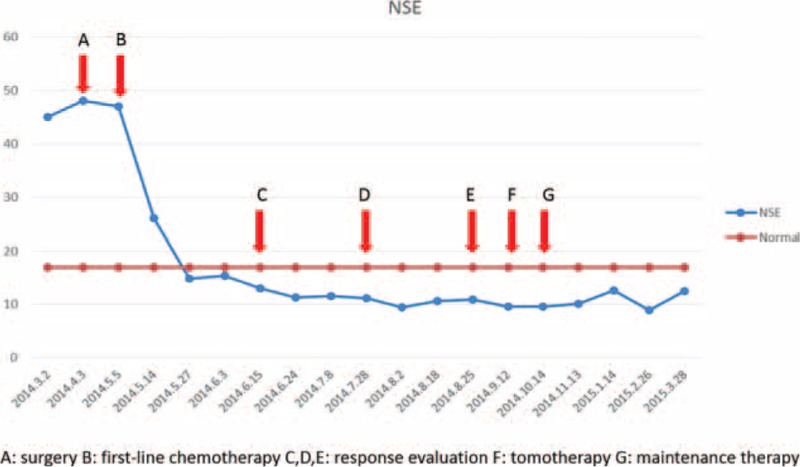
Level of neuron-specific enolase (NSE) decreased gradually during treatment and reached the normal range eventually and persisted.

Because of good response and performance of patient, PMT was adopted with pemetrexed (500 mg/m^2^) administered every 21 days after radiotherapy with another discussion by multidisciplinary team. The interval between the end of radiotherapy and the start of maintenance treatment was 1 month. After 6 courses of PMT, the residual mass disappeared (Figure 3F). The level of NSE decreased gradually during treatment and reached the normal range eventually and persisted (Figure 4). The patient refused further treatment and follow-up started. The patient totally received 6 courses PMT with absence of progression for 14 months from the confirmed diagnosis.

The patient has consented for the publication of the present case report.

## DISCUSSION

MPM is almost always a lethal disease originating from the pleural mesothelium. The prognosis of MPM is poor with median overall survival approximately 1 year and cure is rare. Through this plausible case, this article will discuss some aspects such as whether it is better to add bevacizumab to standard first-line therapy, the benefits of administering maintenance treatment, and the alternative second-line chemotherapy regimens.

### First-Line Treatment of Patients With MPM

In advanced, nonoperable MPM, chemotherapy is still the groundwork of treatment, especially platinum-based regimens. A phase III randomized trial assessed cisplatin/pemetrexed versus cisplatin alone in patients who were not candidates for surgery. The combination of platinum–pemetrexed conferred 3 months median overall survival benefit over cisplatin alone (12.1 months vs 9.3 months, *P* = 0.02).^4^ A combined first-line regimen using cisplatin and pemetrexed is therefore considered as the standard treatment for advanced MPM. Besides, the combination of pemetrexed and carboplatin was also active and well tolerated.^6^

Preclinical studies have clearly suggested that angiogenesis plays an important role in the biology of MPM and that vascular endothelial growth factor (VEGF) and its receptor (VEGFR) are highly presented in MPM.^7,8^ VEGF can stimulate MPM cells proliferation in vitro in a dose-dependent manner and this growth has shown to be inhibited by anti-VEGF antibodies.^9^ Patients with MPM have significantly higher circulating serum VEGF levels than individuals in healthy.^10^ Therefore, antiangiogenic therapies could be effective treatment for patients with MPM.^11^ Bevacizumab is a recombinant humanized monoclonal antibody that blocks the binding of VEGF to its receptor.^12^ So, bevacizumab therapy in MPM may be effective in theory.

The role of bevacizumab adding to the combination of cisplatin and pemetrexed remains indefinite. An open label phase II study (NCT00407459) was to assess the activity of bevacizumab combined with pemetrexed and carboplatin as first-line therapy in patients with MPM.^13^ Unfortunately, however, this trial failed to achieve its primary end point of a 50% improvement in median PFS in comparison with standard pemetrexed/platinum combinations. Excitingly, the final results of the French randomized phase II/III trial (Mesothelioma Avastin cisplatin Pemetrexed Study, MAPS) evaluating the addition of bevacizumab to standard chemotherapy has been reported in 2015 American Society of Clinical Oncology annual meeting.^14^ Bevacizumab addition to pemetrexed/cisplatin provides a significantly longer overall survival (18.82 months vs 16.07 months, *P* = 0.0127) and PFS (9.59 months vs 7.48 months, *P* < 0.001) in advanced MPM patients with acceptable toxicity. The following reasons may account for this reverse results. The former trial has the limitation of a nonrandomized single-arm phase II design that has some potential bias. And this trial also had a crucial limitation of a small sample size (only 76 patients enrolled). While, MAPS is an open-label multicenter randomized trial with restrict inclusion and exclusion criteria. In this trial, 448 patients were included, which contributes to a great statistic power. The results of MAPS trial may be more convinced. The combination of bevacizumab and pemetrexed/cisplatin is very promising and makes this triplet a new treatment paradigm. This triplet may be new standard of fist-line therapy for unresectable MPM.

### Maintenance Treatment of Patients With MPM

Although the response rates in first-line treatment were substantial, most patients experienced disease recurrence or progression during or after first-line treatment.^15^ Limited data are available to guide second-line therapy, although several agents are in clinical trials.^16^ Accordingly, the method of either by prescribing higher dose intensities or higher total doses of chemotherapy may be optimal for patients who are in the absence of disease progression after 4 to 6 cycles of initial therapy. Maintenance chemotherapy, which prolongs duration of chemotherapy either with the use of at least 1 of the agents given in the first line (continuation maintenance) or with the initiation of a different agent (switch maintenance), increases the total dose of chemotherapy.

### Continuation Maintenance Therapy

There existed many clinical investigations to explore the continuation maintenance treatment for patients with MPM. In a small trial with 27 patients, PMT until disease progression or unacceptable toxicity, compared with no maintenance after pemetrexed-based induction treatment, suggested both survival and time to progression benefit (17.9 months vs 6 months and 6 months vs 3.4 months, respectively; *P* < 0.0001)^17^; 23% of patients with stable disease after induction therapy achieved a PR with maintenance therapy. In this investigation, no grade 4 toxicity was observed and the only nonhematological grade 3 toxicity during PMT was fatigue (15%). The safety of continuing single agent pemetrexed has also demonstrated. In addition, pemetrexed as a single agent to treat MPM has revealed activity in a phase II trial. The response rate and median overall survival were 14.1% and 10.7 months, respectively.^18^ On the basis of antitumor activity of pemetrexed, PMT strategy could be available in the current case.

The ongoing randomized CALGB30901 phase II trial, registered on the Clinical Trials website http://clinicaltrials.gov under number NCT00880971, is also assessing the role of pemetrexed as maintenance therapy after an induction with 4 courses of platinum plus pemetrexed. This trial is still recruiting patients and not likely to report final results for another 2 years. In addition, there exist 2 old small trials of maintenance therapy in MPM. One with interferon in which toxicity was tolerate but the effect of maintenance remained unclear.^19^ The other with etoposide administering orally showed that the response status never improved during maintenance treatment.^20^ However, these 2 studies were conducted before the establishment of standard care of first-line chemotherapy. Therefore, these 2 investigations appear outdated and the value of guiding clinical practice is limited.

It is well acknowledged that single-agent bevacizumab^21^ or pemetrexed^22^ is effective as the continuation maintenance therapy in patients with nonsquamous Non-Small Cell Lung Cancer (NSCLC) (who are negative for sensitizing EGFR mutations or Anaplastic Lymphoma Kinase arrangements) beyond 4 to 6 cycles of initial therapy. Dual-agents maintenance therapy using bevacizumab/pemetrexed is also an option in patients with nonsquamous NSCLC.^23,24^ In consideration of good effect with single-agent or dual-agents maintenance therapy for NSCLC, the favorable response may also be presented in MPM. Researchers should launch clinical trials to assess the impact of bevacizumab with or without PMT. It may be difficult for the enrollment of patients as the incidence of MPM is low compared with NSCLC. The final results may be prospective at cost of many years.

### Switch Maintenance Therapy

Continuation maintenance with pemetrexed could achieve a good response, while switch maintenance may be disappointed.

Thalidomide has shown antiangiogenetic activity and immunomodulation by inducing apoptosis of established new vasculature. Besides, it also has a striking bioavailability after oral administration and has shown an outstanding antitumor effect in hematological malignancies.^25–27^ Recently, final results of the NVALT5, an open-label, multicenter, randomized phase 3 study, have been published, which explored thalidomide versus active supportive care for maintenance in patients with MPM after first-line chemotherapy. Patients were randomly assigned to receive thalidomide 200 mg/d plus best supportive care (BSC) or BSC alone until disease progression. Thalidomide did not suggest any positive effect in the maintenance setting in term of time to progression (3.6 months vs 3.5 months, *P* = 0.72) with the addition of thalidomide maintenance to first-line chemotherapy.

In conclusion, the continuation maintenance treatment with pemetrexed may have advantages for patient with MPM, while the switch maintenance treatment with thalidomide yielded disappointing results. The more convenient administration of maintenance therapy in patients with MPM is still an open question. Thereby, further investigations are needed to confirm the effect of maintenance therapy.

### Second-Line Treatment of Patients With MPM

Many patients may want second-line chemotherapy at the time of recurrence or progression. Second-line therapy is being progressively utilized in clinical practice, despite of the fact that there are none appropriate randomized controlled researches to show any survival benefit and the definitive optimal regimen is currently undefined.^16^ The only randomized clinical trial in this setting eliciting an improvement in PFS was undertaken before the widespread use of pemetrexed as first-line treatment, comparing second-line pemetrexed versus BSC.^28^ Pemetrexed alone or in combination with cisplatin could be available as second-line treatment in those patients who previously treated without pemetrexed.^29^ Furthermore, for pemetrexed pretreated patients, the combination of dual-agents treatment with gemcitabine–vinorelbine or gemcitabine–oxaliplatin are reasonable palliative options.^30,31^ Besides, re-treatment with pemetrexed is a feasible option in fit patients, especially in patients with longer PFS (>12 months) after first-line pemetrexed treatment.^32^ The efficacy of second-line therapy remains unanswered at present and it is still an unmet need in this patient population.

## CONCLUSIONS

We described a case of MPM treated by PMT following cisplatin-based bevacizumab-containing first-line chemotherapy, with a perfect response and a long PFS. Although the standard first-line chemotherapy for advanced MPM is the combination of cisplatin and pemetrexed, addition of bevacizumab to standard first-line treatment tend to be favorable. PMT still remains experimental; however, the use of higher total dose of pemetrexed by adopting PMT following first-line treatment appears extremely promising. The administration of maintenance therapy in patients with MPM is still unclear and further investigation is urgently warranted to answer this question.
